# Factors Influencing Surgical Decision-Making in the Posterior Laminectomy With Fixation for Degenerative Cervical Myelopathy (POLYFIX-DCM) Trial: Survey Study

**DOI:** 10.2196/48321

**Published:** 2023-09-12

**Authors:** Stefan Yordanov, Xiaoyu Yang, Oliver Mowforth, Andreas K Demetriades, Marcel Ivanov, Pierluigi Vergara, Adrian Gardner, Erlick Pereira, Antony Bateman, Alexander Alamri, Jibin Francis, Rikin Trivedi, Mark Kotter, Benjamin Davies, Alexandru Budu, POLYFIX-DCM research Collaborative Group

**Affiliations:** 1 Academic Neurosurgery Unit Department of Clinical Neurosurgery Cambridge University Cambridge United Kingdom; 2 Department of Neurosurgery Royal Infirmary Edinburgh Edinburgh United Kingdom; 3 Neurosurgery Department Royal Hallamshire Hospital Sheffield United Kingdom; 4 Department of Spinal Surgery East Suffolk and North Essex NHS Foundation Trust Ipswich United Kingdom; 5 The Royal Orthopaedic Hospital NHS Foundation Trust Birmingham United Kingdom; 6 Department of Neurosurgery St Georges University Hospital NHS Foundation Trust London United Kingdom; 7 Royal Derby Spinal Centre University Hospitals of Derby and Burton NHS Foundation Trust Derby United Kingdom; 8 Department of Neurosurgery Queen Elizabeth Hospital Birmingham United Kingdom; 9 See Acknowledgements

**Keywords:** cervical myelopathy, spondylosis, spondylotic stenosis, disc herniation, ossification posterior longitudinal ligament, degeneration, disability, recovery, questionnaire, decision-making, surgeons, myelopathy, stress, spinal cord, surgeons, surgery, decompression, laminectomy

## Abstract

**Background:**

Degenerative cervical myelopathy (DCM) is estimated to affect 2% of the adult population. DCM occurs when degenerative processes cause compression and injure the spinal cord. Surgery to remove the stress caused by the compression of the spinal cord is the mainstay of treatment, with a range of techniques in use. Although various factors are described to inform the selection of these techniques, there needs to be more consensus and limited comparative evidence.

**Objective:**

The main objective of this survey was to explore the variation of practice and decision-making, with a focus on laminectomy versus laminectomy and fusion in posterior surgery of the cervical spine. We present the results of a survey conducted among the principal investigators (PIs) of the National Institute for Health and Care Research (NIHR) randomized controlled trial on posterior laminectomy with fixation for degenerative cervical myelopathy (POLYFIX-DCM).

**Methods:**

A series of 7 cases were shared with 24 PIs using SurveyMonkey. Each case consisted of a midsagittal T2-weighted magnetic resonance imaging and lateral cervical x-rays in flexion and extension. Surgeons were asked if their preferred approach was anterior or posterior. If posterior, they were asked whether they preferred to instrument and whether they had the equipoise to randomize in the NIHR POLYFIX-DCM trial. Variability in decision-making was then explored using factors reported to inform decision-making, such as alignment, location of compression, number of levels operated, presence of mobile spondylolisthesis, and patient age.

**Results:**

The majority of PIs (16/30, 53%) completed the survey. Overall, PIs favored a posterior approach (12/16, 75%) with instrumentation (75/112, average 66%) and would randomize (67/112, average 62%) most cases. Factors reported to inform decision-making poorly explained variability in responses in both univariate testing and with a multivariate model (R2=0.1). Only surgeon experience of more than 5 years and orthopedic specialty training background were significant predictors, both associated with an anterior approach (odds ratio [OR] 1.255; P=.02 and OR 1.344; P=.007, respectively) and fusion for posterior procedures (OR 0.628; P<.001 and OR 1.344; P<.001, respectively). Surgeon experience also significantly affected the openness to randomize, with those with more than 5 years of experience less likely to randomize (OR –0.68; P<.001).

**Conclusions:**

In this representative sample of spine surgeons participating in the POLYFIX-DCM trial as investigators, there is no consensus on surgical strategy, including the role of instrumented fusion following posterior decompression. Overall, this study supports the view that there appears to be a clinical equipoise, and conceptually, a randomized controlled trial appears feasible, which sets the scene for the NIHR POLYFIX-DCM trial.

## Introduction

Degenerative cervical myelopathy (DCM) is the most common cause of adult spinal cord dysfunction worldwide, estimated to affect 2% of the adult population [1-5]. DCM occurs when degenerative processes cause compression and injure the spinal cord [6-8]. This can lead to a range of disabilities, including imbalance and difficulty walking, loss of manual dexterity, sensory loss, bowel or bladder dysfunction, pain, and in extreme circumstances, paralysis [3,9-11]. Surgery to decompress the spinal cord is the mainstay of treatment [12,13]. International guidelines recommend prompt surgical management to treat moderate to severe or progressive DCM [2,14].

Overall, surgical decompression is recognized to be efficacious; however, the individual gains are highly variable [15-17]. Most patients will make a modest but incomplete recovery [18]. Some will achieve marked recovery, but some will continue to deteriorate. Establishing whether this is determined by surgical technique has been a popular focus of DCM research and remains a topic of debate [19]. Currently, evidence for the superiority of one surgical approach over another is largely equivocal [20-23] or absent, and the choice of surgical procedure is at the discretion of the treating surgeon [24]. Therefore, significant variation in practice is observed worldwide.

This is particularly evident in the decision to supplement posterior decompression with instrumented fusion. Although some routinely offer this option (eg, posterior decompression procedures in the United States increasingly include fusion), others make decisions on a case-by-case basis [21,25-27]. Typically, these are radiological factors, including alignment, number of operated levels, and evidence of mobility. Ultimately, the variation in practice is driven by a paucity of high-quality comparative evidence [28], leading the National Institute for Health and Care Research (NIHR) in the United Kingdom to commission a randomized controlled trial on posterior laminectomy with fixation for DCM (POLYFIX-DCM).

As part of the design process of the POLYFIX-DCM trial, it was important to establish where surgeons had uncertainty or clinical equipoise in the absence of clear evidence. The objective was, therefore, to survey the principal investigators (PIs) of the POLYFIX-DCM trial using a series of clinical cases and to conduct a decision matrix analysis to explore consensus in decision-making.

## Methods

### Procedure

A series of cases were shared with 24 PIs using SurveyMonkey. Each issue consisted of a midsagittal T2-weighted magnetic resonance imaging and lateral cervical x-rays in flexion and extension. The PIs were asked whether their preferred approach was anterior or posterior. If posterior, they were asked whether they preferred to instrument and whether they had the equipoise to randomize in the POLYFIX-DCM trial. This imaging arrangement was selected based on its use by the investigators of the cervical spondylotic myelopathy surgical (CSM-S) trial [29] to review radiological factors deemed essential for surgical decision-making to establish clinical equipoise to randomize in a trial comparing anterior and posterior surgery.

Factors influencing decision-making were obtained from an investigator workshop in concert with the literature. These were radiological factors of alignment, location of compression, number of operated levels, presence of mobile spondylolisthesis, and patient age [30]. To explore these factors, each was categorized: age; location of compression as anterior, posterior, or circumferential; compression levels as single or multiple; and dynamic instability as intersegmental movement between flexion and extension x-rays of at least 3.5 mm [31]; alignment was categorized as either kyphotic or lordotic using the Toyama approach [32]. A line between the posterior-inferior edge of C2 and the superior-posterior edge of C7 was drawn. If the posterior border of C3-6 was behind the bar, it was classed as kyphotic. If the posterior border of C3-6 aligned with the line, it was considered straight, and if it was anterior to the line, it was classified as lordotic [32]. Surgeon demographics, including experience and training background (eg, neurosurgery vs orthopedic surgery), were also considered.

The case vignettes were then developed to enable cross-comparison of these factors. In total, 7 different case vignettes were created (Table 1); 2 cases involved patients aged >75 years; 2 cases had radiological evidence of dynamic instability, including the presence of joint capsule fluid in 1 case. Subluxation on imaging was also present in 2 patients. Choice of cases for the survey might have a bias toward multilevel cases, as evidenced by a mean of 2.75 for compression levels, with a median of 3 (IQR 2-3). Cord signal change on magnetic resonance imaging results was present in 2 cases.

Results were then analyzed using the chi-square test for categorical variables and the Mann-Whitney *U* test for continuous variables. A multivariate model was made using binary logistic regression. Analysis was conducted using RStudio (version 4.1.1), with significance set at *P*<.05.

**Table 1:** Case vignettes.

Case	Age (years)	Age >75 years	Cervical lordosis (lordotic, kyphotic, and straight)	Factor compression (anterior, posterior, and circumferential)	Dynamic instability	Joint fluid	Subluxation	Levels of compression
1	85	Yes	Kyphotic	Circumferential	Yes	No	No	3
2	62	No	Lordotic	Circumferential	No	No	No	4
3	57	No	Straight	Circumferential	Yes	No	No	3
4	70	No	Straight	Anterior	Yes	No	Yes	2
5	68	No	Lordotic	Anterior	Yes	No	Yes	1
6	82	Yes	Straight	Circumferential	No	No	No	2
7	72	No	Kyphotic	Anterior	Yes	Yes	No	3

### Ethics Considerations

The survey was conducted as part of the POLYFIX-DCM Trial, as part of the trial design and assessment of qualitative factors that affect recruitment. The ethics approval number for this study is 21/YH/0253 - Health and Care Research Wales.

## Results

### Overview

**Figure 1:**
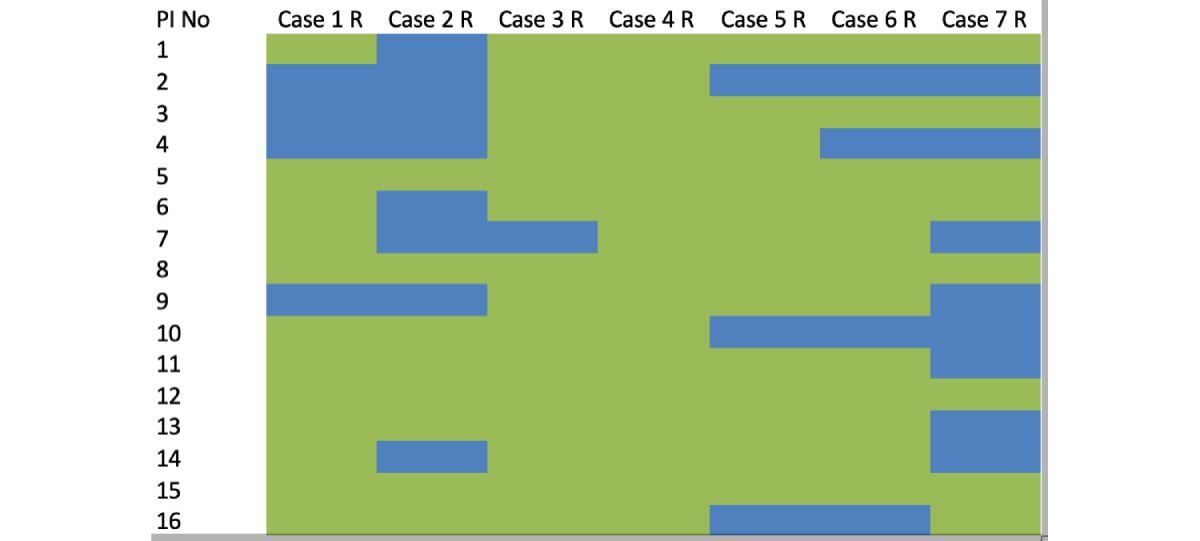
Heatmap showing investigator replies in relation to the chosen surgical approach from the case vignettes. Green represents posterior approach and blue represents anterior approach. No: number; PI: principal investigator.

A total of 16 (53%) of the 30 PIs completed the survey. Most respondents were trained as neurosurgeons (12/16, 75%) and had more than 10 years of experience (Figure 1). After collating the number of PIs (n=16), we explored their decision-making according to each case vignette (n=7), which resulted in 112 entries with problem-specific data. Overall, investigators favored a posterior approach (84/112, 75%) with instrumentation (79/112, 66%) but would randomize most of the cases (67/112, 62%), which is felt sufficient for the purposes of the trial.

### Anterior Versus Posterior Approach

Except for case 4, where all surgeons chose a posterior approach, in the remaining cases, at least three of the 16 surgeons chose an anterior approach. These decisions differed among the same 3 surgeons each time (Figure 1). The predefined factors poorly explained the variability, either through univariate testing (Table 2) or with a multivariate model (*R*^2^=0.1). Only surgeon experience >5 years (odds ratio [OR] 1.255; *P*=.02) and orthopedic training background (OR 1.344; *P*=.007) were significant, both associated with an anterior approach.

**Table 2:** Univariate comparison of factors with surgical decision. Reported values are *P* values. Italicized *P* values denote statistical significance, with *P*<.05.

Factor	Anterior approach	Fusion	Randomize
Dynamic instability	>.99	>.99	>.99
Age	>.99	>.99	>.99
Alignment (kyphotic)	>.99	>.99	>.99
Alignment (straight)	>.99	>.99	>.99
Surgeon background (orthopedic)	*.007* ^a^	*<.001* ^b^	.43^a^
Surgeon experience >5 years	*.02* ^a^	*<.001* ^a^	*.001* ^b^
Circumferential compression	>.99	>.99	>.99

^a^The values signify a significant positive correlation.

^b^The values signify a significant negative correlation.

### To Fuse or Not to Fuse After Posterior Cervical Laminectomy

**Figure 2:**
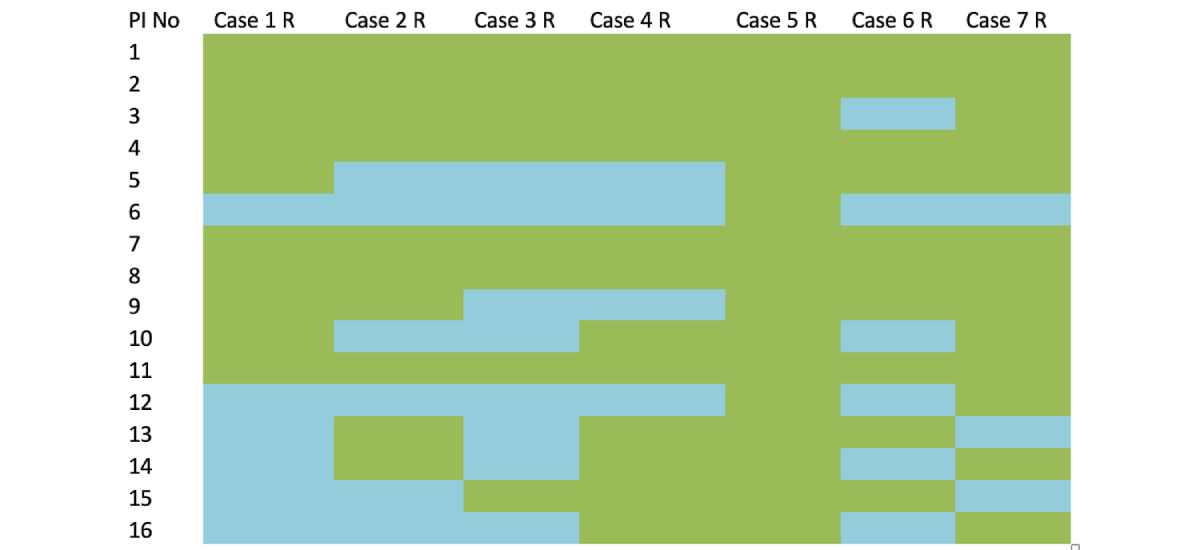
Heatmap showing whether fusion was selected for those preferring a posterior approach. Responses that selected fusion are in green, and those that did not are in blue. No: number; PI: principal investigator.

Except for case 5, where fusion was favored by all surgeons, in all other cases, at least two of the 16 surgeons were in favor of a laminectomy (Figure 2). In total, 5 surgeons would preferentially fuse all cases; however, among the others, there was inconsistent case selection. Although a binary logistic regression model better explained the variability (*R*^2^=0.4), this was exclusively based on surgeon background, with those of orthopedic training (OR 0.628; *P*<.001) and those with an experience of more than 5 years (OR 1.344; *P*<.001) both favoring fusion.

### Randomization Preference if Posterior Approach Is Used

**Figure 3:**
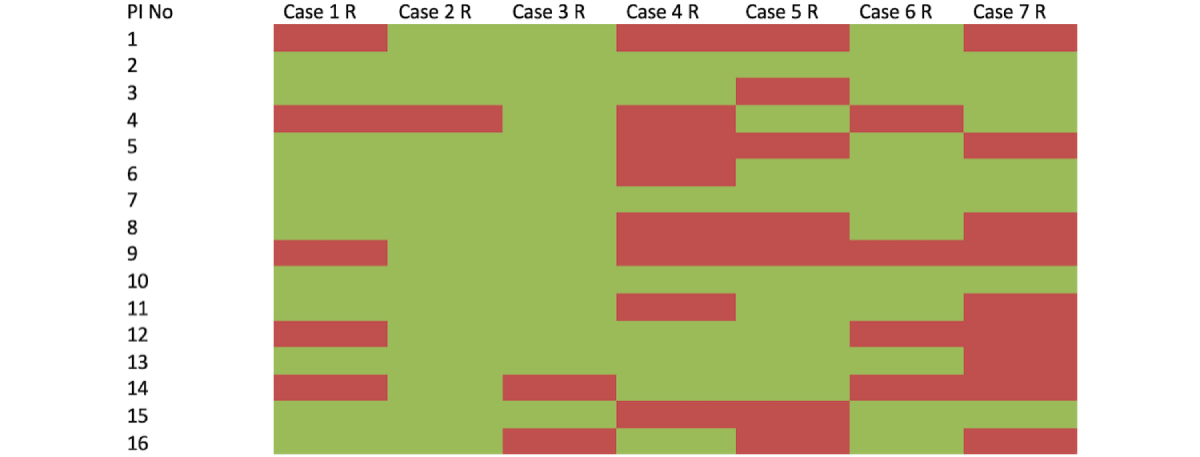
Heatmap showing randomization preferences if a posterior approach is used. PI: principal investigator.

At least seven surgeons were prepared to randomize each case; however, this decision varied by surgeon and case (Figure 3). Exploring this decision-making, the factors involved poorly explained the variability, whether through univariate testing (Table 2) or with a multivariate model (*R*^2^=–0.14). Only surgeon experience significantly affected the openness to randomize, with those with more than 5 years of experience less likely to randomize (OR –0.68; *P*<.001).

## Discussion

### Principal Findings

Surgical decision-making in DCM varied significantly both among surgeons and among cases. This was not necessarily unexpected. Although many factors were proposed to inform decision-making, in this series, these factors could not fully explain the variability among the investigators, and it was impossible to establish specific subgroups. This illustrates both the clinical equipoise and the conceptual feasibility of conducting a randomized controlled trial comparing laminectomy versus laminectomy with instrumented fusion in the United Kingdom.

Furthermore, and most importantly, these findings align with the broader landscape. The premise for the POLYFIX-DCM trial is the absence of high-quality evidence on this topic and an observed variation in practice. The World Federation of Neurosurgical Societies evidence review [27] in 2019 showed in summary that there is no good evidence to suggest any of the treatment modalities. Practice variation in the United Kingdom had principally been measured by surgical activity data, specifically focusing on the proportion of laminectomies versus laminectomies with fusion performed. A criticism of this approach was related to those unmeasured factors, such as alignment or instability, which might help explain the variation of practices. Instead, the factors that showed statistical significance were experience and training background. One hypothesis to explain this phenomenon could be the role of knowledge silos. Knowledge silos are defined as knowledge being centralized and focused within a group of clinicians, usually organizationally or territorially based, that is not being communicated and collaborated across clinician groups or organizations. These are likely significant issues in DCM; for example, fusion is most prevalent in North America compared to laminoplasty in East Asia. The significance of experience also acknowledges the confidence that comes with time, having applied a particular approach successfully.

However, overall, these findings clearly illustrate a clinical equipoise and the necessity for more clarity regarding the circumstances in which fusion should or should not be used. A randomized controlled trial is feasible and should forgo any selection criteria. The NIHR POLYFIX-DCM trial will be the first powered, randomized controlled trial comparing laminectomy versus laminectomy and instrumented fusion for multilevel DCM. The trial hypothesis is that motion restriction through fusion increases neurological recovery. Any patient with moderate to severe DCM or progressive DCM, scheduled for posterior decompression at 2 or more adjacent laminae, is eligible. The primary end point for the trial will be the Modified Japanese Orthopaedic Association at 2 years. Secondary end points of the study are numerous and include quality of life as well as functional and health-economic outcome measures.

Therefore, the NIHR POLYFIX-DCM trial is designed to evaluate routine fusion. Further work is also required to help define potentially significant subgroups if this hypothesis is disproven, to enable planned secondary analysis. Mechanical modelling using Finite Element Analysis is one promising technique [33].

### Limitations

It is essential to acknowledge the limitations of this study. First, many factors aid surgical decision-making, and those selected in this study are not exhaustive. Second, most of the case vignettes focused on multilevel cord compression, which also present a limitation and may have introduced a bias. Third, the survey was only shared among investigators of the POLYFIX-DCM trial, of whom approximately half replied, and this can be an area of potential bias. Consequently, potential areas for improvement are how these findings might generalize as well as whether true results were masked. However, against this are the inclusion of a large proportion (n=24) of UK centers, both orthopedic and neurosurgical. The case vignettes replicated a process, including the factors offered, that was used during randomization by the CSM-S [29] investigators to establish clinical equipoise, and most notably, there was a lack of consistent trends among the data.

Due to the fact that we have surveyed investigators who are part of the POLYFIX-DCM consortium, there is a possibility of inherited bias toward equipoise. We have to be aware that a degree of selection bias could also be present due to the fact that 50% of the surveyed PIs did not respond to our survey.

### Conclusions

In this representative sample of spine surgeons participating as investigators in the POLYFIX-DCM trial, there is no consensus on the role of instrumented fusion following posterior decompression for DCM. Overall, this study supports the view that there appears to be a clinical equipoise, and conceptually, a randomized controlled trial appears feasible, which sets the scene for the NIHR POLYFIX-DCM trial.

## Abbreviations

CSM-S: cervical spondylotic myelopathy surgical
DCM: degenerative cervical myelopathy
NIHR: National Institute for Health and Care Research
OR: odds ratio
PI: principal investigator

## Data Availability

The data sets generated and analyzed during this study are available from the corresponding author on reasonable request.
